# Coccygodynia

**Published:** 1890-03

**Authors:** Hunter P. Cooper

**Affiliations:** Atlanta, Ga.


VOL. VII. MARCH, 1890. NO. 1.

### Original Communications.

### COCCYGODYNIA.*

BY HUNTER P. COOPER, M. D., ATLANTA, GA.

The name Coccygodynia is given to an exceedingly painful affection, dependent upon injury or disease of the coccyx and the sacro-coccygeal nerves. A knowledge of the anatomy of this region will enable us to understand the intense suffering which accompanies the disease. The coccyx, as you know, is the terminal piece of the spinal column, consisting of four rudimentary vertebræ attached to each other so as to form but one bone, and articulating with the tip of the sacrum by an oval concave surface and two small cornua. As it continues the sacral curve its direction is downward with a slight inclination backward. Its posterior surface affords attachment to the great sacro-sciatic ligament and to the gluteus-maximus muscle; to its sides are attached the coccygei, while its anterior surface and apex furnish insertion to the levator-ani and sphincter-ani. On account of its having a single bony articulation it is free to move whenever

*Read before the Atlanta Society of Medicine, February 4, 1890.

the muscles attached to it contract, and lying as it does at the outlet of the pelvis, it feels an effect from each contraction of the muscle of the abdominal walls; it is particularly drawn upon by the contractions of the sphincter-ani, for this muscle has no other bony attachments. The soft parts overlying and surrounding the coccyx are abundantly supplied with sensation by the sacro-coccygeal nerve and numerous filaments from the second, third and fourth sacral nerves.

The disease is more frequently met with in the female than the male, for the reason that child-birth is one of its most frequent causes; it results also from falls or blows or kicks on the coccyx, and occasionally from exposure to cold, as in sitting on a cold, wet seat. Some authors hold that in rare instances it is a pure and simple neuralgia without any traumatic origin; this, however, is doubtful. An injury to the bone, whether from parturition or from external violence, may cause either a fracture or dislocation with considerable deformity. In some cases the injury is followed by caries or necrosis of the bone; in other cases by a chronic inflammation of the periosteum covering the bone; in still other cases no gross pathological changes can be detected, and in these instances it is supposed that the injury has resulted in a morbid excitability of the sacro-coccygeal nerves.

The symptoms of the disease are peculiarly characteristic; they consist of pain in the region of the coccyx, aggravated by all movements of the body which move the coccyx; thus there is pain in sitting down and in rising up, for during the execution of these movements the glutei-maximi muscles contract simultaneously on the two sides, and thus draw the bone strongly forward; again, there is pain in walking, for these same muscles act alternately, and draw the bone first to one side and then to the other. During defecation the levator-ani and sphincter-ani move the coccyx by their contractions and relaxations, and thus frequently cause great pain. While sitting more or less pressure is made on the coccyx, and this proves a frequent source of pain. In a certain proportion of cases the pain which starts in the coccyx extends to the whole of the spine; indeed, the pain in the dorsal or lumbar region may be so severe as to mask the diagnosis and make the physician think he has a case of Pott's disease of the spine to deal with. This was true in the first case I report These symptoms vary in severity in different cases. In some cases the most prominent and distressing symptom is pain in walking; in other cases the greatest pain is felt during defecation; in still others defecation gives no pain whatever, but assuming the sitting posture or rising from the same produces indescribable agony. Pain in defecation was absent in three or four cases that I observed. In some cases the greatest pain is caused by maintaining the sitting posture, and such patients have either to lie down most of the time, or else wear pads over the tuberosities of the ischia in order to take the pressure off the coccyx. Going up or down stairs is painful in a very large proportion of cases.

All sorts of local applications have been used in the treatment of this disease, but without avail. Unless the disease is still in its acute stage, it is a waste of time to try these various measures.

Two operations have been devised for the radical cure of coccygodynia. The first and best of these was described and performed by Dr. Nott, of Mobile, in 1844. It consists in making an incision over the dorsal aspect of the bone, dissecting the soft parts back on each side, dividing the muscular and ligamentous attachments, and disarticulating the bone from the sacrum. During its performance the surgeon should keep his left index finger in the patient's rectum, in order to avoid wounding this organ. The operation is a very easy one, and is usually attended with but slight hemorrhage. The operator can either close the wound up entirely, or, as I prefer, can put in a small drainage tube at the lower angle of the wound.

The other operation was originated by Sir J. Y. Simpson, in 1861, and consists in dividing subcutaneously the muscles and ligaments attached to the coccyx. It is performed by thrusting a tenetone under the skin near the apex of the coccyx and pushing the blade upward until it reaches the upper end of the bone; then it is turned outward and made to divide the structures atached to the lateral border of the bone; the blade is again pushed upward, and the tissues on the other side are divided in a similar manner; the last step in the operation is accomplished by sweeping the knife around the apex of the coccyx and dividing the insertion of the sphincter-ani.

Simpson says: "This simple operation is easy and rapid of performance." Thomas, of New York, says, "I have resorted to this procedure but once, when I found it exceedingly difficult of accomplishment, and it proved an entire failure in giving relief."

I have never tried Simpson's operation, but from my experience in excising the bone, I should say that a complete subcutaneous section of the muscular and ligamentous attachments was well-nigh impossible. As to its efficacy, it often fails to give relief to the symptoms, and the patient must be subjected to a second operation.

In the very case for which Simpson devised it it gave relief for only three or four weeks, when the symptoms returned, and he excised the bone with success.

In the acute stage of the disease, a cure can be often effected by very simple measures. If the symptoms depend upon a recent fracture or dislocation of the coccyx, the physician should introduce his finger into the rectum and reduce the displacement; the patient should be confined closely to bed for at least a week, soothing applications being made over the region of the coccyx. If the case is already several weeks old when the physician first sees it, the use of fly blisters and perfect rest in the recumbent position may cure the disease. If, however, the case is of long standing, nothing short of an operation is apt to afford the sufferer any permanent relief.

CASE I.—Miss A., a healthy, finely developed young lady, received an injury to her coccyx in September, 1884, by sitting down accidentally on the projecting knob of a chair; the pain was at once so severe that she could not sleep at all that night; the following day the pain had subsided sufficiently for her to go around as usual, but each succeeding day she became worse, so that in a week from the time she received the injury, she was compelled to go to bed. She suffered violent pain in the coccygeal region whenever she attempted to walk or to sit up; consequently she was confined to the bed most of the time. It was not long before the pain extended throughout the whole length of her spine. At no time did she suffer pain during defecation.

I saw her in consultation with her physician in February, 1886; she had then been a constant sufferer and invalid for nearly a year and a half, and it was thought by many that she had an incurable disease of the spine. I advised removal of the coccyx, being confident that it was an uncomplicated case of coccygodynia.

The patient went to Savannah in May, 1886, and placed herself in Dr. Elliott's care; she tells me that Dr. Elliott performed three operations on her, the first of which was evidently a subcutaneous division of the tissues according to Simpson's method. (I infer this from the patient's description.) No relief whatever followed this operation, and the next operation was performed in the early summer; this was done with the dental engine, part of the bone being drilled away.

This gave her considerable relief, but she still continued to have pain in walking; for this reason Dr. Elliott removed the whole bone at the third operation. This completely restored her health, and she has remained well ever since.

CASE II.—Mr. D., a merchant, stepped on a loose plank on a platform, and fell, striking his coccyx a violent blow. After suffering greatly for ten days, he consulted me. I found a typical case of coccygodynia in the acute stage. There was neither fracture nor dislocation, but the slightest movement of the bone in any direction produced exquisite pain. A fly blister over the coccyx and perfect rest in bed for one week effected a cure.

CASE III.—Miss C., a young lady from Louisiana, came under my charge the first week in January, 1889, with the following history: She has always been a delicate girl, having had several attacks of malarial fever while living in Louisiana. In April, 1888, she was thrown from her horse, and struck the ground squarely on her buttocks. She was at once taken home and put to bed, suffering great pain throughout the whole length of her spine. She remained in bed for two or three weeks, suffering constant pain in the back, and being utterly unable to stand. There was no impairment of functions of the bladder or rectum. After she got out of bed this spinal pain continued, until in August it began to manifest itself especially in the coccygeal region. The coccyx became the seat of great pain, pain in assuming the sitting posture and in rising from the same, but no pain in defecation; no menstrual derangement occurred. In the autumn all of the symptoms became more aggravated,and now she felt great pain in walking. Her general health began to suffer, she became pale and thin, nervous and sleepless; her whole spine would ache all night long. Even the skin of the back became hyperæsthetic, so that the pressure of clothing produced great discomfort.

Her condition, when I first examined her in January, was as follows: Marked pallor and anæmia, dark rings under eyes, tongue and pulse normal, bowels regular, no appetite; she sleeps only a few hours (2 or 3) each night, and suffers constant pain in the spine from the mid-dorsal region to the coccyx. Pain is aggravated by assuming sitting posture and rising from the same, going up and down stairs, and walking; there is no pain in defecation or afterwards. Menstruation is normal in every way, except she is more nervous at such times. She complains very little, and manifests no hysteria whatever. Examination of spine shows normal contour, no lateral curvature, and perfect flexibility. Tenderness on pressure is very marked over spinous processes from mid-dorsal region to tip of coccyx. The skin on each side of the spinal column is hyperæsthetic. She cannot allow any thorough examination of the coccyx, because of the intense pain it causes. There is, however, no perceptible deviation of the coccyx from its normal position.

Treatment: I determined to exhaust all the simpler measures before resorting to an operation. I put her on a tonic containing pyrophos. iron, quinine and strychnia, and at the same time began the use of a mild descending galvanic current, one pole at nape of neck, the other at sacrum; these séances were repeated every other day for four weeks without causing any improvement; at the end of that time I determined to try the effect of absolute rest, so she went to bed and for three weeks scrupulously maintained the recumbent position. During the first two weeks of this time she improved considerably, but in the third week her appetite failed completely, her insomnia grew worse, and her pain returned with increased violence. I then thought of trying the effect of the actual cautery along her spine, but as the hyperæsthesia of the skin was so marked, I first applied a fly blister, with the idea of resorting to the cautery later, if the blister should have a beneficial effect. The blister intensified the spinal pain greatly, rather than relieving it, and it took a full week for the patient to return to her former condition. Her suffering from the blister was so great that I abandoned the idea of using the cautery. About this time it became impossible for her to sit down squarely on account of the pain in the coccyx. I called Dr. Hardon in consultation; he found no evidence of hysteria, and thought the symptoms did not warrant a vaginal examination. He suggested the local use of iodine to the whole length of the spine and the internal use of Donovan's Solution. This plan was persisted in until the first of April without any visible improvement, when I got Dr. Baird to see her with me. He advised the administration of a pill containing quinine, iron, strychnia, arsenic and gentian. The continued use of this improved her appetite and strength somewhat, but her suffering was in no degree lessened.

April 21st. After taking some exercise, she was taken with a very severe pain in the coccyx, which rapidly increased in severity, so that when she reached home it was really unbearable, and I was hastily summoned. I have never seen a patient with renal or hepatic colic suffer more terribly; morphine hypodermically in large and repeated doses, and chloral and potass. bromide internally had no effect whatever on the pain, so that I was forced to give her inhalations of chloroform; after keeping her under the influence of chloroform more or less for an hour, the morphine began to exert its effect, but it was several hours before she was completely relieved.

May 8th. Since the last note she has had three distinct attacks just as severe as the one described above. I called Drs. Hardon, Noble and Baird in consultation, and after etherizing the patient, each of these gentlemen made a thorough vaginal examination to ascertain whether there was any uterine disease which could explain her symptoms. All of them concurred in the opinion that there was no disease of the pelvic organs. I therefore proceeded with their approval to excise the coccyx. This was done, and the wound stitched with cat-gut, a short drainage tube being placed in the lower angle of the wound.

May 11th. Healing by first intention is taking place. Patient has suffered less pain since the operation than she has in months.

May 14th. Continued improvement. Normal temperature since last note. Wound has healed entirely, except where the drainage tube is. The old pain is entirely gone.

May 24th. Patient has not suffered any pain since last note until to-day; I was called to see her and found her in one of her old attacks, though the pain was not nearly so intense; the wound is still suppurating at the point where the drainage tube was inserted, but everywhere else it healed by first intention.

June 12th. In the last two weeks the patient has had six attacks of spinal pain, each attack being less severe than the one preceding it. All of them except one were controlled without the use of morphine.

July 1st. She has been sitting up for several days; eats well and sleeps well without any medicine. She has had no pain since June 11th, and is rapidly gaining flesh and strength. The wound is completely healed, and all the hyperæsthesia has disappeared from the spine.

July 17th. Patient's general condition has continued to improve; she has had no pain whatever, walks up and down stairs with impunity, and sits in an uncushioned chair without discomfort. Discharged cured.

Soon after this she took a trip north with my consent, for I thought the change would do her good. But in that I was mistaken, for she had a return of the pain, evidently brought on by the railroad journey, and ever since then she has suffered with her back, though the pain has never been as severe as before the operation. I attribute her relapse to the fact that not only the coccyx, but the whole spine, had been injured, and the pain returned principally above the seat of operation.

CASE IV.—Mrs. H., a married lady, 28 years old, came under my charge August 15th, 1889, with the following history: Ever since the birth of her last child (about six years ago) she has suffered with marked symptons of coccygodynia. On examination I found the coccyx tilted sharply backward out of its normal position, and also deflected to the right. Any pressure on the bone or movement of it was attended with very severe pain. It was evident that the coccyx had been dislocated backward during the passage of the child's head through the outlet of the pelvis, and had become more or less firmly anchylosed in this new position.

With the assistance of Drs. Hardon and Harris, I removed the coccyx on the following day, and the patient made a rapid recovery, the whole wound healing by first intention, except where the drainage tube was inserted. The operation relieved her pain very considerably, but before she had time to fully realize the benefits of the operation, she underwent another operation for a bilateral laceration of the cervix uteri; she made an excellent recovery from this also, and when the stitches were removed, the union was found to be perfect; unfortunately on the very day the stitches were taken out, she disobeyed her physician's orders, got out of bed and walked around the room. This imprudence was followed by a severe attack of pelvic cellulitis, which confined her to bed for a number of weeks, and from the effects of which I fear she will never entirely recover. Following this attack, the pain in her back returned to a certain degree, but not nearly so severe as before the operation.

The failure to effect a perfect cure in these two last cases was due, I think, to the existence of a concomitant injury of the spine in the one case, and to the presence of cellulitis in the other.

A curious motor disturbance appeared in two of the cases; in Case I, there was considerable disability of one of the lower extremities, on account of a peculiar "drawing" of the limb, as the patient described it. In Case III, there was for two months after the operation a marked loss of co-ordination during locomotion, and as a consequence the patient would wobble from side to side somewhat after the manner of a drunken man. It may be that these symptoms depend upon the removal of Luschka's gland, a small ganglion of the sympathetic situated directly in front of the coccyx.

